# Synthesis of a resin monomer-soluble polyrotaxane crosslinker containing cleavable end groups

**DOI:** 10.3762/bjoc.10.274

**Published:** 2014-11-10

**Authors:** Ji-Hun Seo, Shino Nakagawa, Koichiro Hirata, Nobuhiko Yui

**Affiliations:** 1 Institute of Biomaterials and Bioengineering, Tokyo Medical and Dental University, Tokyo, 101-0062, Japan; 2 Tokuyama Dental Corp. Research Institute, Tsukuba, 300-4247, Japan

**Keywords:** α-cyclodextrin, composite resin, disulfide, polyrotaxane, Vickers hardness

## Abstract

A resin monomer-soluble polyrotaxane (PRX) crosslinker with cleavable end groups was synthesized to develop degradable photosetting composite resins. The PRX containing 50 α-cyclodextrins (α-CDs) with disulfide end groups was initially modified with *n*-butylamine to obtain a resin monomer-soluble PRX. The PRX containing 13 *n*-butyl groups per α-CD molecule was completely soluble in conventional resin monomers such as 2-hydroxyethyl methacrylate (HEMA) and urethane dimethacrylate (UDMA). The synthesized *n*-butyl-containing PRX was further modified with 2-aminoethyl methacrylate to provide crosslinkable acrylic groups onto PRX. The prepared resin monomer-soluble PRX crosslinker was successfully polymerized with a mixture of HEMA and UDMA to provide photosetting plastic. It was confirmed that the Vickers hardness of the prepared plastic was greatly decreased after treatment with dithiothreitol. This indicates that the resin monomer-soluble PRX crosslinker can be applied to design degradable photosetting plastics potentially used in the industrial or biomedical field.

## Introduction

Polyrotaxane (PRX) is a supermolecule containing host molecules, e.g., α-cyclodextrin (α-CD), threaded on a linear guest molecule, e.g., poly(ethylene glycol) (PEG) [1]. The threaded α-CD molecules are known to be reversibly disassembled when a cleavable end-capping group was introduced in the PEGs, and the cleavable reaction was triggered by proper signals [2–4]. For example, if cleavable end-capping groups such as disulfide groups are introduced at both ends of PRX, the threaded α-CD molecules could be completely released by disassembling the PRX structure when a preferable stimulation such as dithiothreitol (DTT) is applied to the cleavable end-capping groups [5–6]. Because this assembling–disassembling process could be induced by a minimal chemical reaction, i.e., a single end-group reaction for the entire polymer chain, the induction of the disassembling process in the case of α-CD molecules is much more effective than in the case of other types of macromolecular assemblies or stimuli-degradable polymer networks [7–9]. For this reason, various types of cleavable PRXs have been designed for the development of controlled release systems of specific molecules [10]. A representative example is the application as a protein or gene delivery system. Aminated PRXs with cleavable disulfide end groups can form stable complexes with negatively charged biomacromolecules such as proteins, pDNA, or siRNA [11–15]. These PRX–biomolecule complexes were successfully delivered into the cytoplasm, and the biomolecules were effectively released after the disassembling of PRX by a cytoplasmic reductant such as glutathione. The characteristic property of cleavable PRXs may also be harnessed to develop a stimuli-responsive crosslinker to modulate the hardness of acrylic resins. Acrylic resins are irreversible photosetting polymers adopted to increase the mechanical strength in various applied polymer materials used in industrial or biomedical fields [16–17]. For example, composite resins containing 2-hydroxyethyl methacrylate (HEMA) or methyl methacrylate (MMA) have been widely used to increase the mechanical strength of restorative or adhesive materials in dentistry [18]. The rapid polymerization of acrylic groups with crosslinkable dimethacrylate monomers such as triethyleneglycol dimethacrylate (TEGDMA) or urethane dimethacrylate (UDMA), and the high permeability of the resin monomers to dental tissue have been suggested as optimal properties to develop strong and stable prosthetic materials [19]. Although an increased strength of composite resins is preferable to develop prosthetic materials, this increased strength is often considered unfavorable when the polymerized composite resins need to be removed after the treatment. In this kind of application, the introduction of a stimuli-responsive and degradable crosslinkable resin monomer into the composite resin, the hardness of the polymerized resin composite could be modulated under specific conditions. Because the degradable PRX structure is a suitable molecular platform for an efficient degradation with minimal chemical reaction, methacrylate-functionalized PRX with degradable end-capping groups is anticipated to be a useful molecular platform for the development of degradable crosslinking agents. In this study, resin monomer-soluble, methacrylate-functionalized PRX with degradable end-capping groups is synthesized for modulating the hardness of composite resins.

## Results and Discussion

**Scheme 1:**
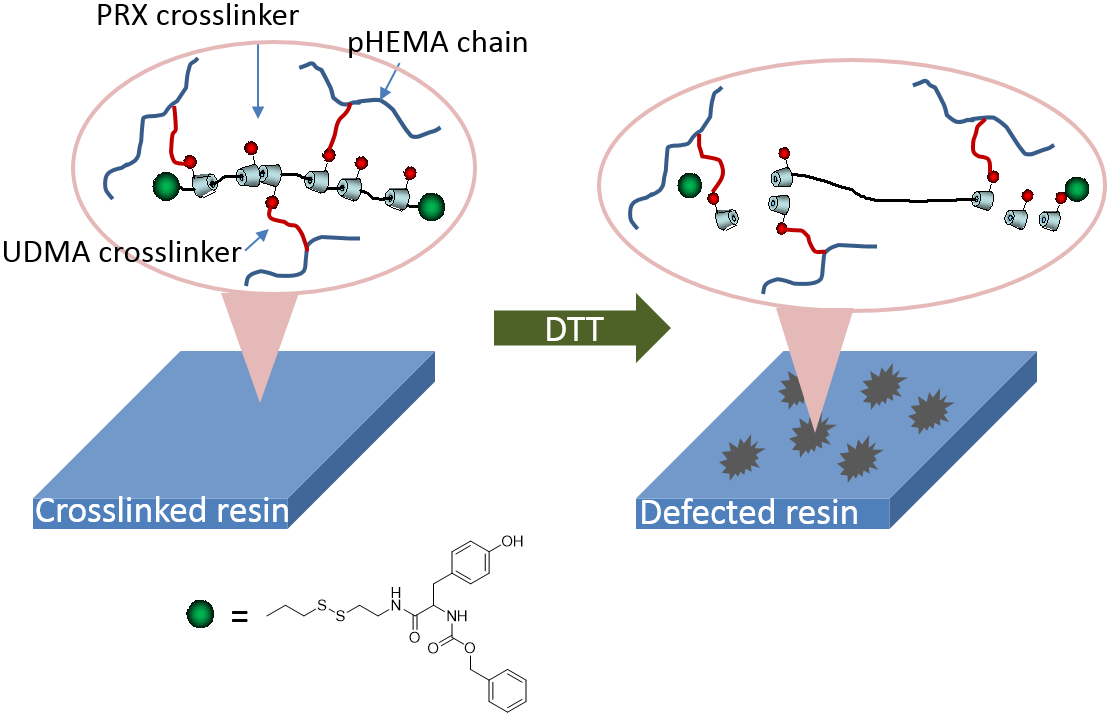
Overall concept of the degradable PRX crosslinker.

**Scheme 2:**
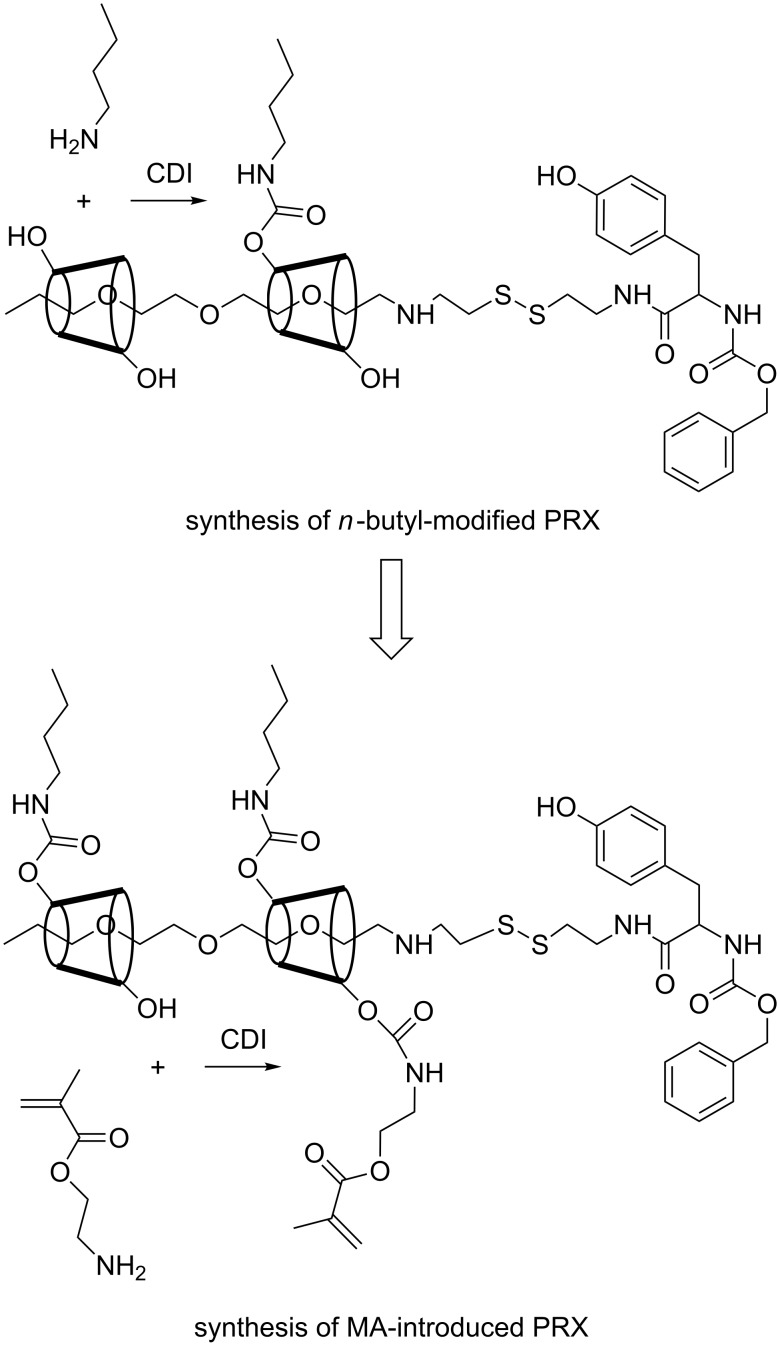
Overall reaction scheme of the resin monomer-soluble PRX crosslinker with degradable end groups.

Scheme 1 shows the concept of modulating the hardness of photosetting polymer networks by means of degradable PRX crosslinkers. Because the degradable nature could be induced by a mild end-group reaction, PRX might be a useful molecular platform in such an application. The most critical step in the preparation of PRX-containing composite resins is to synthesize resin monomer-soluble PRX derivatives. Generally, PRX derivatives show poor solubility in various organic solvents, including liquid resin monomers, due to a large number of hydroxy groups on α-CDs [20]. Therefore, research work has been conducted to enhance the solubility of the PRX-based materials in a variety of solvents [21–23]. In the present study, PRX derivatives soluble in resin monomers, i.e., a mixture of HEMA and UDMA, were synthesized. A PRX containing ca. 50 CDs with disulfide end groups was synthesized as previously reported [11]. The prepared PRX was only soluble in dimethyl sulfoxide (DMSO), and it showed poor miscibility with liquid resin monomers such as HEMA or UDMA. In order to increase the miscibility with the resin monomers, the hydroxy groups of α-CDs were modified by *n*-butyl groups by using carbonyldiimidazole (CDI) (Scheme 2).

Because the α-CD molecule contains 18 hydroxy groups, 18 butyl groups could be theoretically introduced per α-CD molecule. The degree of control of the substitution of hydroxy groups was investigated by changing the in-feed ratio of *n*-butylamine and CDI. The resulting compositions of *n*-butyl-modified PRX are summarized in Table 1.

**Table 1:** Molecular profiles of the *n*-butyl-modified PRXs.

Symbol	In feed (molar ratio to α-CD)		In polymer (^1^H NMR)	Solubility with resin monomer (50 wt % of PRX)	

	CDI	*n*-butylamine	*n*-butyl/α-CD (yield, %)	HEMA	UDMA

C3-Bu12	3	12	3.4 (78)	X	X
C6-Bu12	6	12	5.2 (72)	X	X
C12-Bu12	12	12	13.1 (82)	O	O
C6-Bu24	6	24	6.0 (75)	X	X
C12-Bu24	12	24	13.2 (85)	O	O

**Figure 1:**
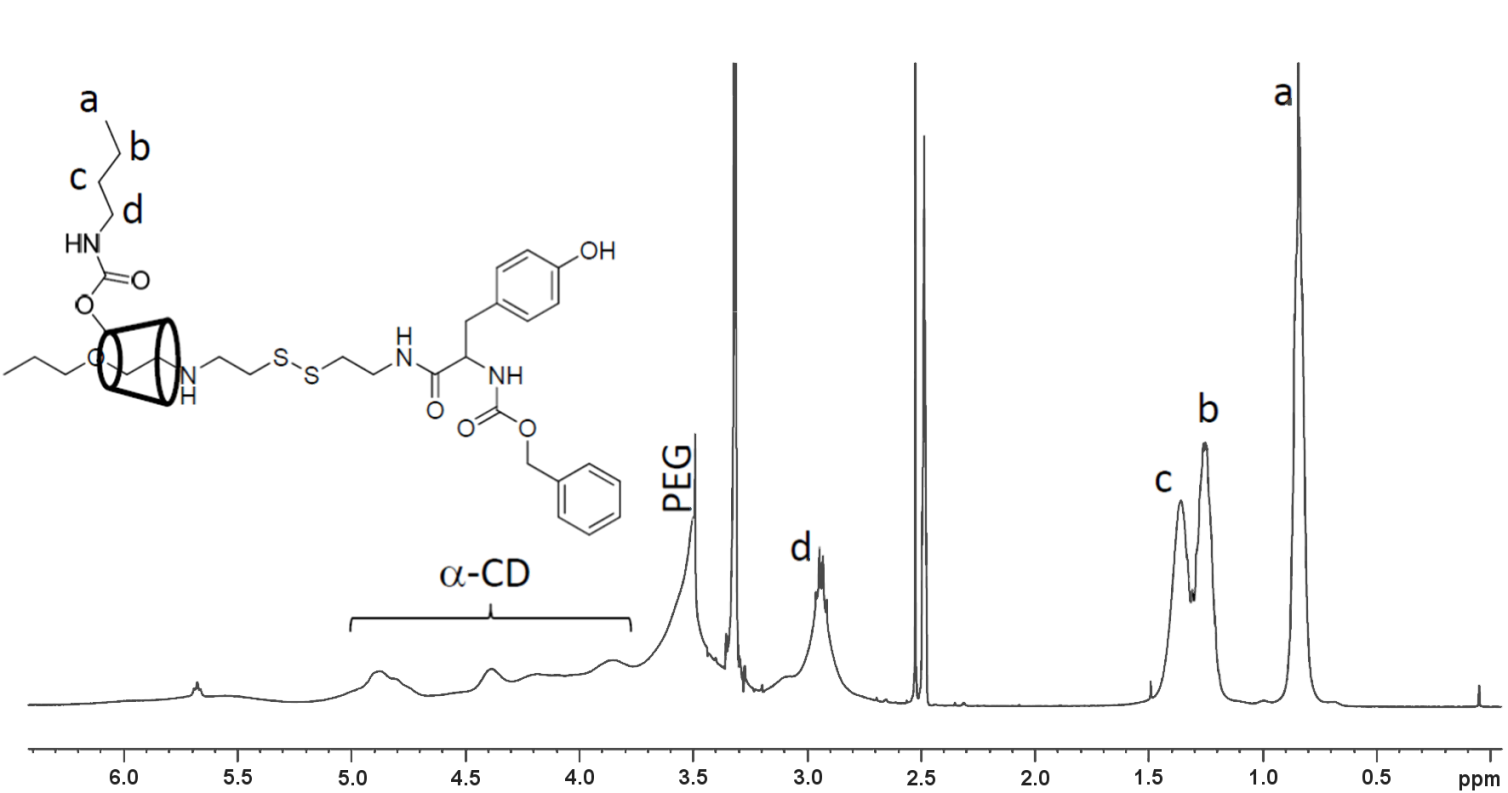
^1^H NMR spectrum of C12-Bu12 PRX (DMSO-*d*_6_).

The substitution of hydroxy groups with *n*-butyl groups was effectively controlled by changing the in feed of CDI rather than changing the *n*-butylamine. The number of *n*-butyl groups per α-CD in synthesized PRX was possibly slightly higher than the in-feed ratio due to the inevitable calculation error induced by the broadening of the α-CDs peak after the chemical modification in ^1^H NMR as shown in Figure 1. In any event, we could successfully control the composition of *n*-butyl groups to examine the solubility of the synthesized PRX with respect to the resin monomers. Figure 1 shows the representative ^1^H NMR spectrum of *n*-butyl-modified cleavable PRX (C12-Bu12), and strong signals of *n*-butyl group are observed within 0.5–1.5 ppm.

**Figure 2:**
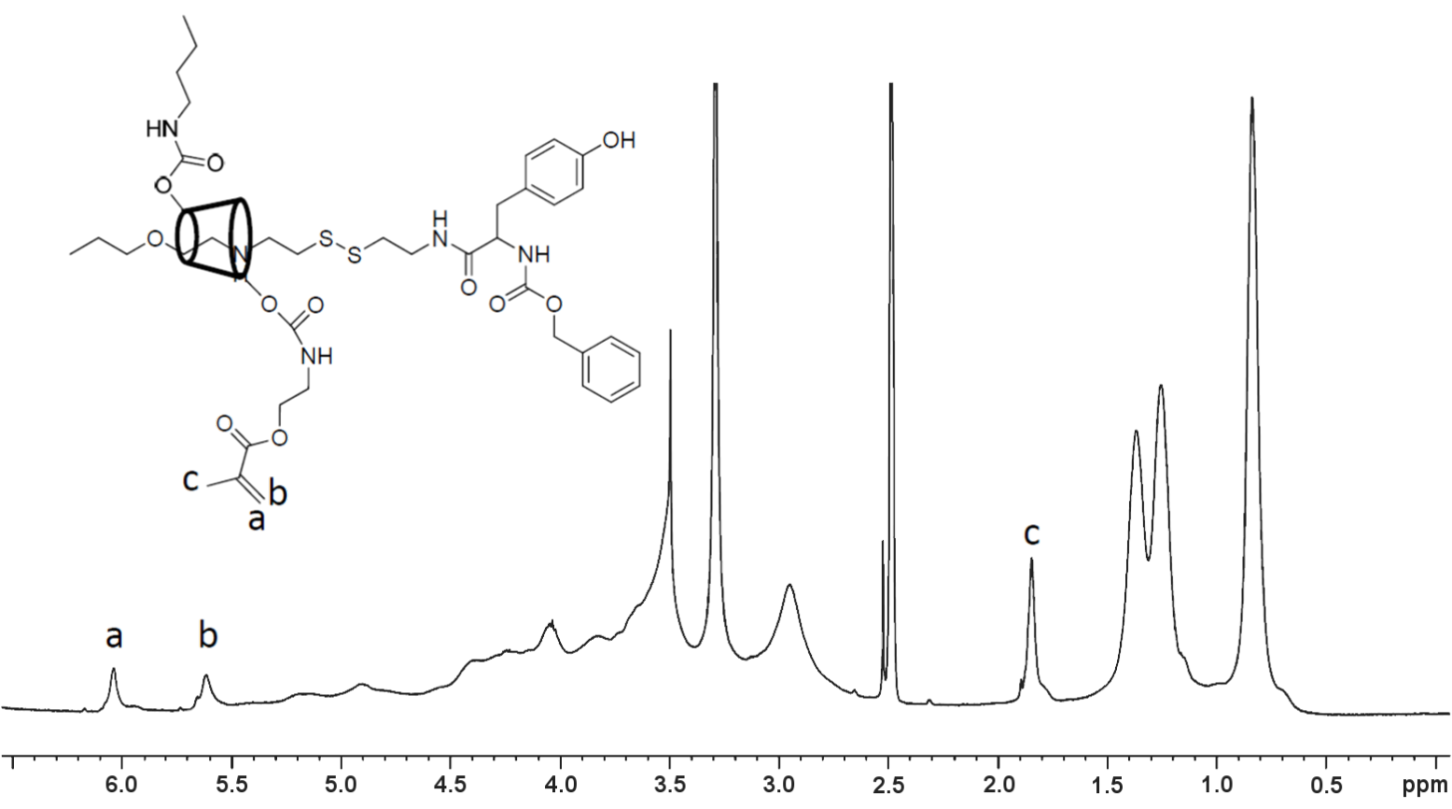
^1^H NMR spectrum of C12-Bu12-MA12 PRX (DMSO-*d*_6_).

The modified PRXs were then mixed (50 wt %) with HEMA or UDMA monomers, respectively, and the resulting optical transparency is listed in Table 1. As a result, clear mixtures of the *n*-butyl-modified PRXs with resin monomers were obtained when the number of introduced *n*-butyl groups was ca. 13 per α-CD molecule. This indicates that C12-Bu12 or C12-Bu24 is an optimized composition to homogeneously mix the degradable PRX with conventional resin monomers. The synthesized C12-Bu12 PRX was further allowed to react with 2-aminoethyl methacrylate to provide methacrylate groups, which could be crosslinked to the resin monomer-soluble PRXs (Scheme 2). Table 2 shows the reaction conditions and the resulting composition of methacrylate (MA) in *n*-butyl-modified PRXs. The number of MA groups per α-CD was limited to 0.91 even under conditions of excess CDI or 2-aminoethyl methacrylate monomer. Because C12-Bu12 PRXs contain 13 *n*-butyl groups per α-CD molecule, densely introduced *n*-butyl groups may hinder the residual hydroxy groups to be further modified with 2-aminoethyl methacrylate monomers. Figure 2 shows the ^1^H NMR spectrum of the C12-Bu12-MA12 PRX, and MA group signals are confirmed. As a result, it was confirmed that ca. one MA group per α-CD molecule could be introduced on C12-Bu12 PRX, and this MA group-containing PRX also showed good miscibility with HEMA and UDMA.

**Table 2:** Molecular profiles of methacrylate-functionalized PRXs.

Symbol	In feed (molar ratio to α-CD)		In polymer (^1^H NMR)	Solubility with resin monomer (50 wt % of PRX)	

	CDI	2-aminoethyl methacrylate (MA)	MA/α-CD (yield, %)	HEMA	UDMA

C12-Bu12-MA6	6	6	0.58 (92)	O	O
C12-Bu12-MA12	6	12	0.91 (92)	O	O
C12-Bu12-MA-24	6	24	0.91 (98)	O	O

**Figure 3:**
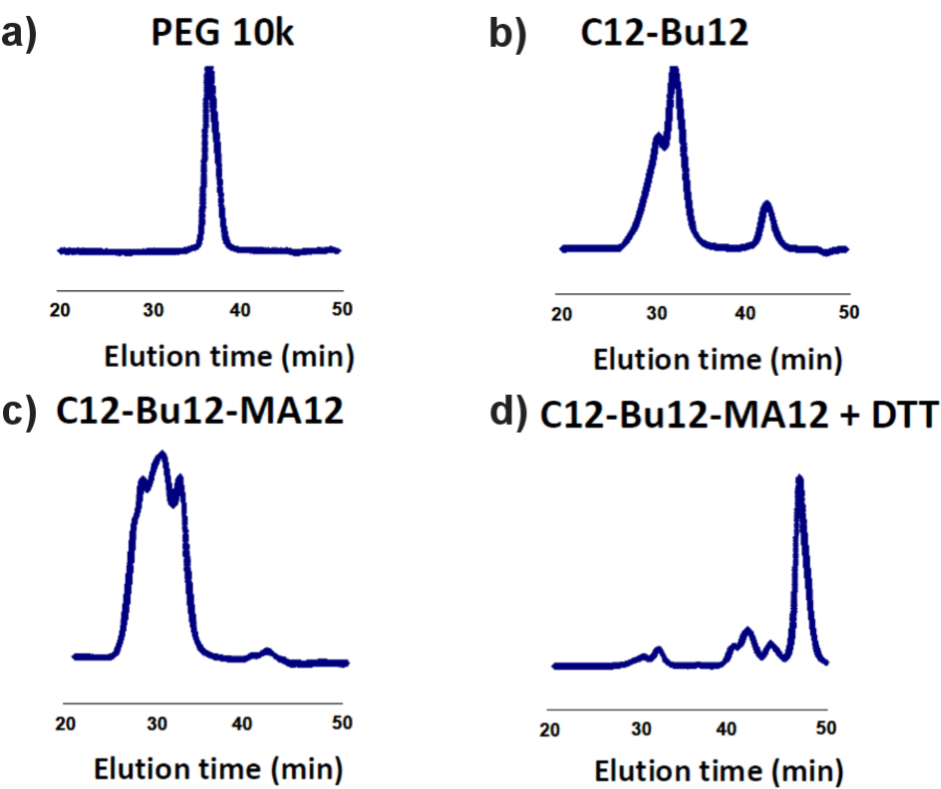
SEC analysis of the prepared polymers a) PEG 10 k, b) C12-Bu12, c) C12-Bu12-MA12, d) C12-Bu12-MA12 + DTT.

The degradable nature of the synthesized PRX (C12-Bu12-MA12) was confirmed by size-exclusion chromatography (SEC) before and after the treatment with DTT. Figure 3 shows the results of the SEC analysis of the synthesized polymers. Although a slight degradation of PRX was observed (disassembled α-CD at 42 min), C12-Bu12 and C12-Bu12-MA12 show shorter elution times compared to PEG, which indicates that the molecular weight is increased due to the formation of an inclusion complex of PEG with ca. 50 α-CDs. The degradation nature of C12-Bu12-MA12 was confirmed after the treatment with DTT. As shown in Figure 3, the PRX SEC signal almost disappeared, and a large intensity of low molecular weight signals was observed within 40–50 min. The low molecular weight signals observed within 40~45 min are probably induced by disassembled α-CD molecules. Because the degree of the modification of α-CDs could be different among the individual α-CDs, multiple signals are possibly shown for α-CDs. The sharp signal shown around 48 min may be caused by the residual DTT in the solution. In any event, it could be confirmed that C12-Bu12-MA12 is completely degraded by the cleavage of the disulfide end groups, and a large number of α-CD molecules are released.

**Figure 4:**
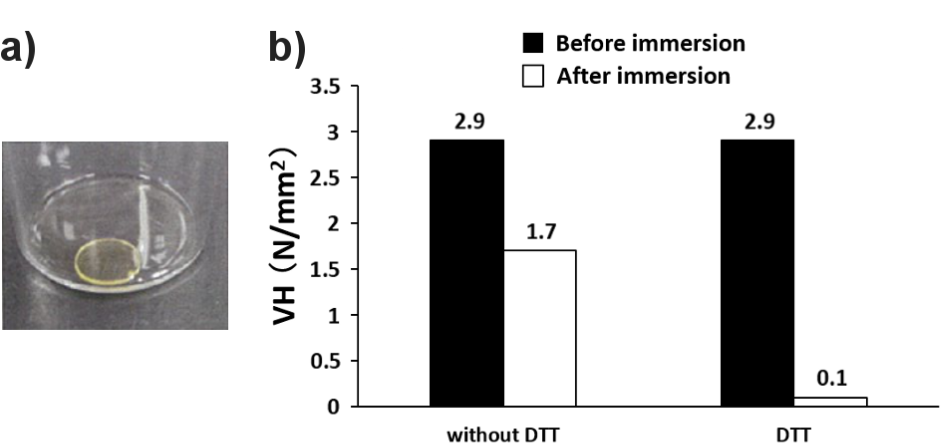
a) Image of the photosetting plastic prepared by resin casting and b) the results of the Vickers hardness (VH) measurement before and after the treatment with DTT.

Synthesized C12-Bu12-MA12 was then polymerized with HEMA and UDMA via camphorquinone-initiated polymerization. Figure 4 shows the image of the polymerized mixture prepared by resin casting.

As shown, slightly yellow and transparent plastic was formed, indicating that the well-soluble PRX crosslinker does not result in a heterogeneous aggregation during the polymerization process. The effect of degradable crosslinkers on the change of hardness was estimated by means of the Vickers hardness test. As shown in Figure 4, the initial Vickers hardness of the hardened plastic was 2.9 N/mm^2^. Generally, conventional dental composite resin shows around 15 N/mm^2^ [24]. Therefore, the prepared PRX-based thermosetting plastic exhibits a lower hardness than conventional resin, which may be caused by the high composition of PRX, i.e., the decreased amount of resin monomers. The optimization process of the enhanced hardness is currently under investigation, and a discussion about the initial hardness is out of the scope of this study. The sample was then immersed in acetone (without DTT) for three days and naturally dried for further analysis. After the immersion the Vickers hardness decreased to 1.7 N/mm^2^ (i.e., to 58.6% of the initial hardness of 2.9 N/mm^2^). This is probably due to the decrease in the density of the hardened sample because non-crosslinked polymer chains or residual monomers might be eluted when the sample was immersed in acetone. In order to confirm the effect of the degradable PRX crosslinker on the change in hardness, the sample was immersed in acetone containing DTT and naturally dried for further analysis. Figure 4 also shows the result of the Vickers hardness measurement of the DTT-treated sample. The Vickers hardness drastically decreased to 0.1 N/mm^2^ (to 3.45% of the initial hardness 2.9 N/mm^2^). This significant decrease in the hardness is thought to be due to the disruption of the polymer network by the degraded crosslinker. Because the single C12-Bu12-MA PRX chain contains ca. 45 MA groups, C12-Bu12-MA PRX is anticipated to act as a strong crosslinker during the polymerization. After being immersed in DTT-containing acetone, the strongly formed polymer network might be disrupted to a significant extent due to the cleaved disulfide end groups of PRX. This is thought to be the reason of the drastic decrease in the hardness of the photosetting plastic.

Photosetting plastic is widely used in the field of dental treatment as a form of a composite resin. The mechanical strength of the polymerized composite resin is strong enough to preserve the prosthetic devices. However, the simple removal of the crosslinked complex resin after treatment has been an urgent problem in the field of dentistry. In this study, we could suggest a cleavable supramolecular crosslinker which is miscible with resin monomers. We expect that the present concept could be applicable to design a stimuli-cleavable composite resin in the field of dental treatment.

## Conclusion

The optimized molecular structure of a degradable PRX crosslinker was examined in order to develop a crosslinker soluble in conventional resin monomers. The optimized PRX crosslinker was highly soluble in conventional resin monomers, and the hardness of polymerized plastic could be successfully modulated by inducing the degradation of the PRX crosslinker. This molecular concept could be applied to design degradable photosetting plastics which may potentially be utilized in the industrial or biomedical field.

## Experimental

### Materials

A PRX containing 50 α-CDs with disulfide end groups was synthesized by using PEG 10k as previously reported in [11]. *n*-Butylamine and camphorquinone were purchased from Tokyo Kasei Co. (Tokyo, Japan). 2-Aminoethyl methacrylate hydrochloride, *N*,*N*’-carbonyldiimidazole, ethyl 4-(dimethylamino)benzoate (DMBE), 3,5-di-*tert*-butyl-4-hydroxytoluene (BHT) and 2-hydroxyethyl methacrylate were purchased from Sigma-Aldrich Chemical Co. (St. Louis, MO, USA). Urethane methacrylate (Art Resin^TM^) was purchased from Negami Chemical Industrial Co., Ltd. (Ishikawa, Japan). All the organic solvents used in the present study were purchased from Kanto Chemical Co.(Tokyo, Japan) and used as received.

### Synthesis of *n*-butyl-modified PRX

A representative synthetic procedure for *n*-butyl-modified PRX is as follows. Initially, 300 mg of PRX were dissolved in dry DMSO. To this solution, 488 mg of CDI (3.0 mmol, at a molar ratio of 1:12 with respect to α-CD) was added and stirred at rt for 3 h. Then, 220 mg of *n*-butylamine (3.0 mmol, at a molar ratio of 1:12 with respect to α-CD) was added to the solution, and stirred at rt for 3 d. The reaction mixture was then transferred to a dialysis tube (MWCO 8k) and dialyzed in methanol for 2 d. The methanol solution was then dropped into 50 mL of water, and the white, heterogeneous water suspension was centrifuged at 2000 rpm for 10 min. The obtained precipitate was dispersed in 5 mL of water and freeze-dried to obtain the white polymer product.

### Synthesis of methacrylate-functionalized PRX

A representative synthetic procedure is as follows. 150 mg of C12-Bu12 PRX were dissolved in 3 mL of dry DMSO. To this solution, 122 mg of CDI (at a molar ratio of 1:6 with respect to α-CD) were added and stirred at rt for 3 h. Then, 248 mg of 2-aminoethyl methacrylate hydrochloride (at a molar ratio of 1:12 with respect to α-CD) were added and stirred at rt for 3 d. The reaction mixture was then transferred to a dialysis tube (MWCO 8k), and dialyzed in methanol for 2 d. The methanol solution was then dropped into 50 mL of water, and the white, heterogeneous water suspension was centrifuged at 4000 rpm for 10 min. The obtained precipitate was dispersed in 5 mL water and freeze-dried to obtain white polymer product.

### Preparation of degradable photosetting plastic

A mixture of 25 wt % of HEMA and 75 wt % of crosslinker (50 wt % C12-Bu12-MA12 PRX and 25 wt % UDMA) with 5 wt % (to total monomer mixture) of initiator mixture (camphorquinone 2 wt %, CMBE 2 wt %, BHT 1 wt %) was dissolved in 200 wt % acetone and cast in a 8 mm in diameter and 0.5 mm deep disk-type Teflon assembly mold and dried by air blowing. The mold was covered with slide glass, and the mixture was photo-polymerized for 20 s with Elipar^TM^ S10 curing light (wavelength ~455 nm, 3M ESPE, St. Paul, MN, USA). The prepared photosetting plastic was then immersed in 1 mL acetone (with or without 0.5 M DTT) and aged for 3 d to investigate the degradable nature of prepared photosetting plastic.

### Characterization

^1^H nuclear magnetic resonance (NMR) measurement was conducted with a Bruker Avance III 500 MHz spectrometer (Bruker Biospin, Rheinstetten, Germany) in DMSO-*d*_6_ (Sigma-Aldrich). Size-exclusion chromatography (SEC) analysis was conducted by using a JASCO RI-1530 detector containing two connected TSK-GEL, α-4000 and α-2500 gel columns (Tosoh Corp. Tokyo, Japan) with DMSO (10 mM LiBr) as an eluent at 37 °C.

The Vickers hardness was measured by using PMT-X7A (Matsuzawa Co., Akita, Japan). In particular, a diamond indenter was loaded (F = 5 gf) onto the sample for 30 s, and the crack image was analyzed to measure longitudinal and transverse axes of the indent (*d*_1_ and *d*_2_). Vickers hardness was calculated by means of the equation VH = 2 × F × sin(θ/2)/*d*^2^, with *d* = (*d*_1_ + *d*_2_)/2, where θ is the angle of the cross axes of the diamond indenter.
